# Aerobic and resistance exercise training reverses age‐dependent decline in NAD^+^ salvage capacity in human skeletal muscle

**DOI:** 10.14814/phy2.14139

**Published:** 2019-06-17

**Authors:** Roldan M. de Guia, Marianne Agerholm, Thomas S. Nielsen, Leslie A. Consitt, Ditte Søgaard, Jørn W. Helge, Steen Larsen, Josef Brandauer, Joseph A. Houmard, Jonas T. Treebak

**Affiliations:** ^1^ Novo Nordisk Foundation Center for Basic Metabolic Research Faculty of Health and Medical Sciences University of Copenhagen Copenhagen Denmark; ^2^ Department of Biomedical Sciences Ohio Musculoskeletal and Neurological Institute Diabetes Institute Ohio University Athens Ohio; ^3^ Xlab Center for Healthy Aging Department of Biomedical Sciences University of Copenhagen Copenhagen Denmark; ^4^ Clinical Research Centre Medical University of Bialystok Bialystok Poland; ^5^ Department of Health Sciences Gettysburg College Gettysburg Pennsylvania; ^6^ Department of Kinesiology Human Performance Laboratory East Carolina University Greenville North Carolina; ^7^ East Carolina Diabetes and Obesity Institute East Carolina University Greenville North Carolina; ^8^ Present address: Department of Medicine Laval University Quebec QC Canada

**Keywords:** Aging, exercise training, NAD^+^ salvage pathways, NAMPT, skeletal muscle

## Abstract

Aging decreases skeletal muscle mass and strength, but aerobic and resistance exercise training maintains skeletal muscle function. NAD ^+^ is a coenzyme for ATP production and a required substrate for enzymes regulating cellular homeostasis. In skeletal muscle, NAD ^+^ is mainly generated by the NAD ^+^ salvage pathway in which nicotinamide phosphoribosyltransferase (NAMPT) is rate‐limiting. NAMPT decreases with age in human skeletal muscle, and aerobic exercise training increases NAMPT levels in young men. However, whether distinct modes of exercise training increase NAMPT levels in both young and old people is unknown. We assessed the effects of 12 weeks of aerobic and resistance exercise training on skeletal muscle abundance of NAMPT, nicotinamide riboside kinase 2 (NRK2), and nicotinamide mononucleotide adenylyltransferase (NMNAT) 1 and 3 in young (≤35 years) and older (≥55 years) individuals. NAMPT in skeletal muscle correlated negatively with age (*r* ^2^ = 0.297, *P* < 0.001, *n* = 57), and VO_2_peak was the best predictor of NAMPT levels. Moreover, aerobic exercise training increased NAMPT abundance 12% and 28% in young and older individuals, respectively, whereas resistance exercise training increased NAMPT abundance 25% and 30% in young and in older individuals, respectively. None of the other proteins changed with exercise training. In a separate cohort of young and old people, levels of NAMPT, NRK1, and NMNAT1/2 in abdominal subcutaneous adipose tissue were not affected by either age or 6 weeks of high‐intensity interval training. Collectively, exercise training reverses the age‐dependent decline in skeletal muscle NAMPT abundance, and our findings highlight the value of exercise training in ameliorating age‐associated deterioration of skeletal muscle function.

## Introduction

In humans, aging impairs multiple biological processes from a molecular to organismal level, and affects mental and cardio‐respiratory health (North and Sinclair 2012), body composition (Bischof and Park 2015), and muscle function (Curtis et al. 2015). The loss of skeletal muscle mass with aging in the absence of disease results in decreased muscle strength (Hughes et al. 2015) and increased susceptibility to injury (Baker 2017). Deregulated nutrient sensing, genomic instability, and mitochondrial dysfunction are other hallmarks of aging (Lopez‐Otin et al. 2013).

Regular physical activity has antiaging effects and reduces the risk of injuries in older individuals (Montero‐Fernandez and Serra‐Rexach 2013; Aguirre and Villareal 2015). Moreover, muscle morphology, fiber size, and functional properties including strength, are preserved by lifelong physical activity (Zampieri et al. 2015). Both aerobic and resistance exercise training exert multiple beneficial effects on the aging body (Vopat et al. 2014; Borde et al. 2015; Milanovic et al. 2015; Mancini et al. 2017). Endurance aerobic exercise training, as well as high‐intensity interval training (HIIT), improves VO_2_max irrespective of age (Borde et al. 2015; Milanovic et al. 2015). Of clinical relevance for the aging population, resistance training improves skeletal muscle strength substantially in older adults (Borde et al. 2015). The molecular mechanisms by which older humans maintain musculoskeletal function are of emerging interest, particularly in developed nations with aging populations.

Sirtuins (SIRTs) are protein deacylases with longevity‐promoting functions (Imai and Guarente 2014). Aging‐associated processes like mitochondrial biogenesis (Canto and Auwerx 2009), oxidative stress (Merksamer et al. 2013), circadian rhythm (Masri and Sassone‐Corsi 2014), and brain function (Satoh et al. 2017) are regulated by SIRTs. An important aspect of sirtuin activity is their dependency on NAD^+^ as a substrate (Imai et al. 2000; Min et al. 2001). Besides supporting sirtuin actions, NAD^+^ is an indispensable molecule for cellular survival. For example, NAD^+^ is an essential redox molecule used for ATP production and it is a substrate for poly‐ADP ribose polymerases (PARPs), which are involved in repairing DNA damage (Burkle 2006). NAD^+^ is also important for the generation of Ca^2+^‐mobilising second messengers (Guse 2015). In both humans and rodents, NAD^+^ concentrations and sirtuin activity reportedly decline with aging in a tissue‐specific manner (Braidy et al. 2011; Massudi et al. 2012). Diminished sirtuin activity in human skeletal muscle may impair oxidative capacity and mitochondrial function, and lead to sarcopenia (Conley et al. 2000; Short et al. 2005).

**Figure 1:**
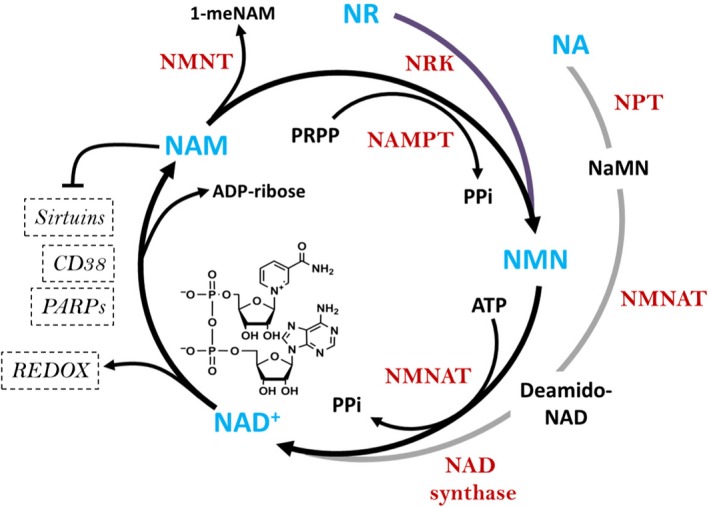
NAD^+^ salvage pathway in mammals. NAM is the major NAD^+^ precursor in mammals. NA and NR can also be used to synthesize NAD^+^. NAMPT is the rate‐limiting step for the synthesis of NAD^+^ from NAM. Once NMN from NR and NAM or deamido‐NAD from NA are formed, it is converted to NAD^+^ by the action of NMNAT or NAD synthetase, respectively. The NAD^+^ generated can then be used for cellular redox reactions or as substrate for the activity of PARPs and sirtuins. NA, nicotinic acid; NAD^+^, nicotinamide adenine dinucleotide; NAMPT, nicotinamide phosphoribosyltransferase; NAM, nicotinamide; NR, nicotinamide riboside; NRK, nicotinamide riboside kinase; NPT, nicotinic acid phosphoribosyltransferase; NMN, nicotinamide mononucleotide; NMNAT, NMN adenylyltransferase; NNMT, nicotinamide‐N‐methyltransferase; NaMN, nicotinic acid mononucleotide; PRPP, phosphoribosyl pyrophosphate; ATP, adenosine triphosphate; PPi, inorganic pyrophosphate; PARPs, poly‐ADP ribose polymerases.

Nicotinamide phosphoribosyltransferase (NAMPT) catalyzes the rate‐limiting step in the intracellular salvage of NAD^+^ from nicotinamide (NAM) (Fig. 1). NAMPT also exists in an extracellular form (eNAMPT) (Tanaka et al. 2007). NAMPT is a ubiquitously expressed protein, and whole‐body knockout of *Nampt* in mice is embryonically lethal (Revollo et al. 2007; Zhang et al. 2017). Knockdown of *Nampt * in C2C12 cells reduces NAD^+^ and decreases maximal respiratory capacity (Agerholm et al. 2018). Muscle‐specific *Nampt* knockout mice exhibit an 85% decrease in intramuscular NAD^+^ levels, which subsequently causes muscle fiber degeneration and progressive loss of muscle strength and endurance capacity (Frederick et al. 2016). NMNAT exists in three isoforms (NMNAT1‐3) that are mostly ubiquitously expressed (Brazill et al. 2017).

An alternative NAD^+^ precursor is nicotinamide riboside (NR) (Bunprajun et al. 2013). NR is converted to NMN by NR kinases (NRK) 1/2 (Fig. 1) (Bieganowski and Brenner 2004; Ratajczak et al. 2016). Dietary supplementation of NR in rodents increases NAD^+^ levels in skeletal muscle (Canto et al. 2012), and improves skeletal muscle and muscle stem cell function in mice with aging or muscular dystrophy (Ryu et al. 2016; Zhang et al. 2016). The potential effects of NR in human skeletal muscle are unknown, but dietary supplementation of NR in humans increases NAD^+^ and NAD^+^‐related metabolites in plasma, peripheral blood mononuclear cells, and urine (Trammell et al. 2016; Dellinger et al. 2017; Dollerup et al. 2018; Martens et al. 2018).

The present study builds on previous reports identifying aerobic exercise training as a potent stimulus to increase NAMPT levels in human skeletal muscle (Costford et al. 2010; Brandauer et al. 2013; Johnson et al. 2015). However, evidence is lacking regarding the potency of distinct modes of exercise training to increase NAD^+^ salvage enzyme levels in skeletal muscle and adipose tissue of young and older humans. To this end, we assessed the age‐dependent effect of 12 weeks of either aerobic or resistance exercise training on skeletal muscle abundance of NAD^+^ salvage enzymes (i.e., NAMPT, NRK, and NMNATs). In light of evidence suggesting that the ability to generate NAD^+^ by NAMPT in adipose tissue is important for adipose function (Nielsen et al. 2018), and clinical studies showing that acute aerobic exercise induces *NAMPT* expression in human subcutaneous abdominal adipose tissue (Frydelund‐Larsen et al. 2007), we also determined whether the abundance of NAD^+^ salvage pathway enzymes are affected by age or HIIT in biopsies of human abdominal subcutaneous adipose tissue.

Here we provide evidence that various exercise training modalities completely correct the age‐dependent decline in skeletal muscle NAMPT abundance. Conversely, neither age nor exercise training affect levels of adipose tissue NAD^+^ salvage enzymes. Our findings underscore the importance of regular physical activity to restore skeletal muscle NAD^+^ salvage capacity with age and have general implications for treatment of metabolic disease.

## Methods

### Ethics approval

Study participants gave their written informed consent prior to their participation (Consitt et al. 2013; Larsen et al. 2015; Søgaard et al. 2017). Study protocols were in accordance with the guidelines of the Declaration of Helsinki II and approved by the East Carolina University Policy and Review Committee on Human Research (*Exercise training – muscle samples*) and by the Ethical Committee of Copenhagen and Frederiksberg (#H‐3‐2012‐024: *Exercise training – adipose tissue samples*).

### Exercise training – muscle samples

Skeletal muscle samples from 57 individuals comprising a wide age range (18–84 years) were obtained from a previous study with a total of 73 participants (Consitt et al. 2013). The reason for only including 57 individuals was due to lack of sample material from 16 individuals. The physical and physiological characteristics of the 57 individuals are provided in Table 1. Data summarized in Table 1 were used for correlation analyses with skeletal muscle levels of NAMPT and plasma eNAMPT abundance. From the 57 subjects, 43 individuals participated in an exercise training study where people aged ≤ 35 years (*n* = 21) were classified as “young” and those aged ≥ 55 years (*n* = 22) as “older”. The exercise training programs consisted of 12 weeks of aerobic exercise (treadmill, stationary cycle, or elliptical trainer) or 12 weeks of resistance exercise (upper and lower body exercises). For the aerobic training, a target heart rate zone equivalent to 70–75% of VO_2_peak was set for a total of 180 min/week (3–4 sessions per week). For the resistance exercise training, alternating upper and lower body exercises were performed for ~45 min/session (3 sessions per week). Resistance was increased by 5% when subjects could complete 12 repetitions on two consecutive occasions. Muscle biopsies were collected before initiation of the training program (baseline) and 40 h after the final training session (post‐training). The biopsies were obtained under local anesthesia from the *vastus lateralis* with the percutaneous muscle biopsy technique.

**Table 1:** Participant characteristics from cross‐sectional study

	Men	Women	ALL	*n*_Total_
Age	44.4 ± 4.8 (27, 19–84)	48.5 ± 3.6 (30, 18–76)	46.5 ± 3.0	57
Mass (kg)	81.9 ± 2.3 (27, 64.5–102.7)	70.3 ± 2.1 (30, 55–91.4)^a^	75.8 ± 1.7	57
BMI (kg/m^2^)	25.8 ± 0.8 (27, 20–32)	26.2 ± 0.7 (30, 19–31)	26 ± 0.5	57
Body fat (%)	23.3 ± 1.9 (27, 14–39)	38.9 ± 1.5 (30, 16–53)^a^	31.5 ± 1.6	57
Trunk fat (%)	28.0 ± 2.4 (24, 15.9–45.1)	39.8 ± 1.7 (30, 14.3–54.8)^a^	34.6 ± 1.6	54
Lean body mass (kg)	58.2 ± 1.1 (27, 48.3–68)	39.6 ± 1.0 (30, 31.7–47)^a^	48.2 ± 1.4	57
Waist‐to‐hip ratio	0.87 ± 0.02 (26, 0.76–1.04)	0.76 ± 0.01 (29, 0.66–0.92)^a^	0.81 ± 0.01	55
VO_2_peak (mL/kg/min)	34.4 ± 2.4 (26, 12.6–58.7)	25.0 ± 1.4 (30, 12.6–37.9)^a^	29.3 ± 1.5	56
Fasting plasma glucose (mg/dL)	89.0 ± 1.5 (27, 73–105)	88.5 ± 1.9 (30, 71–118)	88.7 ± 1.2	57
Fasting plasma insulin (*μ*LU/mL)	5.8 ± 0.7 (27, 1.5–12.9)	6.8 ± 1.1 (30, 1.5–15.7)	6.3 ± 0.65	57
HOMA‐IR	1.29 ± 0.16 (27, 0.27–2.88)	1.58 ± 0.30 (30, 0.26–7.94)	1.4 ± 0.17	57
*M*‐value	7.1 ± 0.55 (27, 2.22–14.11)	7.5 ± 0.45 (30, 3.01–14.67)	7.31 ± 0.35	57
Blood cholesterol (mg/dL)	181.4 ± 8.8 (27, 125–262)	189.8 ± 6.4 (30, 135–269)	185.8 ± 5.3	57
HDL cholesterol (mg/dL)	47.7 ± 2.0 (27, 33–71)	56.1 ± 2.1 (30, 41–80)^a^	52.1 ± 1.5	57
LDL cholesterol (mg/dL)	107.7 ± 6.5 (27, 59.8–154)	111.8 ± 5.6 (30, 72.4–177)	109.9 ± 4.3	57
Blood trigylcerides (mg/dL)	130.0 ± 17.0 (27, 48–357)	109.5 ± 8.3 (30, 63–246)	119.2 ± 9.1	57

Data are presented mean ± SEM (*n*, range).

a *P* < 0.05 versus men.

### Exercise training – adipose tissue samples

Adipose tissue samples from 30 individuals (22–74 years) were obtained from two previous studies (Larsen et al. 2015; Søgaard et al. 2017). In brief, participants underwent HIIT on a bicycle ergometer three times per week for 6 weeks. Each HIIT session had five 1‐min intervals interspersed with 1.5 min rest. Biopsies were obtained under local anesthesia from the abdominal, subcutaneous adipose tissue using the Bergstrom needle technique.

### Western blot analysis

Tissue samples were homogenized using steal beads (Tissue Lyser II, Qiagen) in ice‐cold lysis buffer at pH 7.4. Muscle samples were homogenized in a buffer containing 10% glycerol, 1% IGEPAL, 50 mmol/L Hepes, 150 mmol/L NaCl, 10 mmol/L NaF, 1 mmol/L EDTA, 1 mmol/L EGTA, 20 mmol/L sodium pyrophosphate, 2 mmol/L sodium orthovanadate, 1 mmol/L sodium‐pyrophosphate, 5 mmol/L nicotinamide, 4 *μ*mol/L Thiamet G, and protease inhibitors (SigmaFast, Sigma Aldrich). Adipose tissue biopsies were homogenized in a buffer containing 20 mmol/L Hepes, 10 mmol/L NaF, 1 mmol/L sodium orthovanadate, 1 mmol/L EDTA, 5% SDS and protease inhibitors (SigmaFast, Sigma Aldrich). BCA assays (Thermo Scientific, #23223 and #23224) were used to quantify protein concentration. The same amount of protein was loaded into each well which, depending on the antibody, ranged from 15 to 20 *μ*g. Proteins were resolved by SDS‐PAGE and transferred to polyvinylidene difluoride (PVDF) membranes (Millipore, Denmark) as described (Brandauer et al. 2013). Western blots were conducted in a balanced design, with samples from all training conditions present on all gels, and identical internal controls included on each blot. The internal control was prepared as a pool of all samples. Four to five trimmed gels were transferred to the same PVDF membrane. Individual samples were normalized to the same internal control loaded on each gel in order to permit comparison of samples resolved on separate gels. Following transfer, membranes were incubated with the following primary antibodies: NAMPT (Bethyl, A300‐779A), NRK1 (Santa Cruz, SC‐398852), NRK2 (Santa Cruz, SC‐240683), NMNAT1 (Abcam, 45548), NMNAT2 (Adipogen, AG‐20A‐0087), NMNAT3 (Abcam, 116228). NAMPT antibody was optimized and used as described (Brandauer et al. 2013). NRK1, NRK2, NMNAT1‐3 antibodies were optimized for the linear range of detection. Recombinant proteins were used to validate antibody specificity. After primary antibody incubation, membranes were washed and incubated with peroxidase‐conjugated secondary antibody. Western blots were visualized using Biorad ChemiDoc chemiluminescence system, and densitometry analyses were performed using ImageLab software (Biorad, Hercules, CA, USA). For adipose tissue samples, proteins were loaded and resolved in Criterion TGX Stain‐Free precast gels (Bio‐Rad). The gel was then activated for 1 min and photographed using the ChemiDoc Touch Imaging System. Total stain‐free fluorescence and signal intensity for the specific antibody were analyed using ImageLab software. The total stain‐free fluorescence lane volume was used for normalization (Gurtler et al. 2013).

### Extracellular NAMPT quantification

Extracellular NAMPT levels before and after exercise training were quantified from plasma of 39 out of 43 participants in the exercise training studies using an ELISA according to manufacturer's instructions (Adipogen, AG‐45A‐0006EK‐KI01). Standards and plasma samples (100 *μ*L) were analyzed in duplicates.

### Statistics

Data are reported as mean ± SEM. Statistical analyses were performed by 2 × 2 repeated‐measures analysis of variance (ANOVA). The Tukey test was used post hoc. Strength of association between variables was measured using Pearson correlation coefficient, *r*, for data following Gaussian distribution, and the nonparametric Spearman correlation coefficient, *r*_s_, for data sets not passing normality test (*α* = 0.05). *P*‐values were corrected for multiple hypothesis testing using Benjamini–Hochberg algorithm and false discovery rate (FDR) set at 0.05 for all tests. Potential predictors identified for skeletal muscle NAMPT abundance were analyzed by multiple stepwise regression analysis. Data on age, lean body mass, triglycerides, insulin, HOMA‐IR, and waist‐to‐hip ratio were transformed to meet the assumption of normality. Data were handled and analyzed using Microsoft Excel (Microsoft, WA, USA), GraphPad Prism 7 (GraphPad Software, Inc., CA, USA), and IBM SPSS Statistics (IBM, NY, USA).

## Results

### Subject characteristics: “Muscle cohort”

A subset of individuals from a previous study (Consitt et al. 2013) was used to measure NAD^+^ salvage‐related protein abundance in skeletal muscle. In this subset of participants, there were gender differences in body weight, lean mass, waist‐to‐hip ratio, VO_2_peak, body and trunk fat percentage, and HDL cholesterol levels (Table 1). Moreover, age correlated positively with BMI (*r* ^2^ = 0.2279, *P* < 0.001), body fat percentage (*r* ^2^ = 0.301, *P* < 0.001), waist‐to‐hip ratio (*r* ^2^ = 0.202, *P* < 0.001), and fasting blood glucose (*r* ^2^ = 0.181, *P* < 0.001) while VO_2_peak was negatively correlated with age (*r* ^2^ = 0.628, *P* < 0.001).

### Skeletal muscle NAMPT abundance correlates with distinct physiological parameters

**Figure 2:**
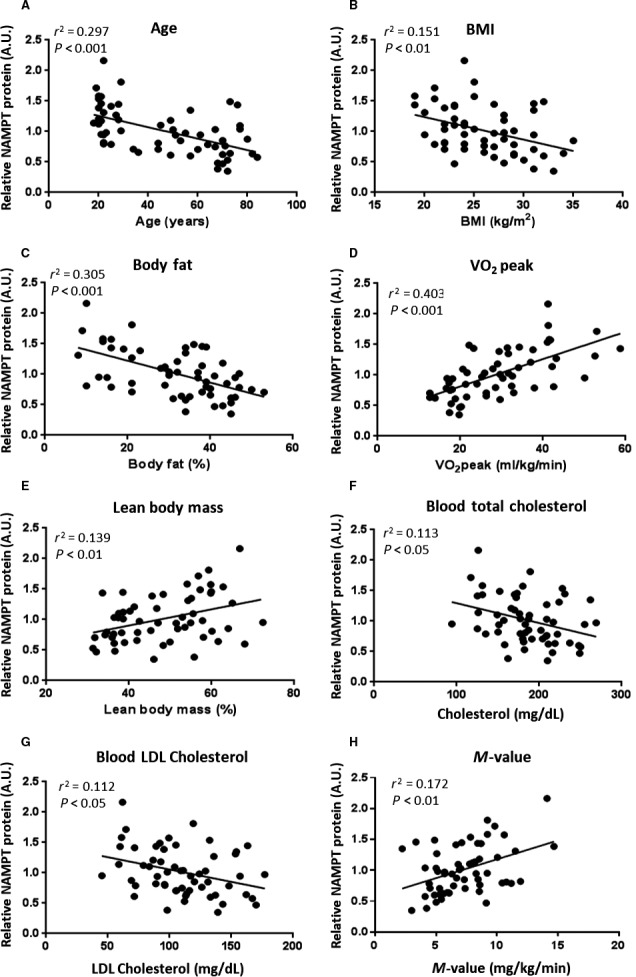
NAMPT protein levels in human skeletal muscle declines with increasing age and body fat. Correlation of the baseline skeletal muscle NAMPT with A, age (*n* = 57, *r* ^2^ = 0.297, *P* < 0.001)**; B, BMI (*n* = 57, *r* ^2^ = 0.151, *P* < 0.01)*; C, body fat (*n* = 57, *r* ^2^ = 0.305, *P* < 0.001)*; D, VO_2_peak (*n* = 57, *r* ^2^ = 0.403, *P* < 0.001)*; E, lean body mass (*n* = 57, *r* ^2^ = 0.139, *P* < 0.01)**; F, total blood cholesterol (*n* = 57, *r* ^2^ = 0.113, *P* < 0.05)*; G, blood LDL cholesterol (*n* = 57, *r* ^2^ = 0.112, *P* < 0.05); H, *M*‐value (*n* = 57, *r* ^2^ = 0.172, *P* < 0.01)*. *Pearson correlation coefficient, *r* was used. Significance of Pearson coefficient was tested using the *t*‐distribution. **Spearman correlation coefficient, *r*_s_ was used.

Correlation analyses were used to determine whether baseline skeletal muscle NAMPT protein levels correlated with age and other parameters measured in the study. Protein levels of NAMPT correlated negatively with the age of the participants (*r* ^2^ = 0.297, *P* < 0.001; Fig. 2A). Furthermore, negative correlations were found between NAMPT and BMI (*r* ^2^ = 0.151, *P* < 0.01; Fig. 2B), and between NAMPT and body fat percentage (*r* ^2^ = 0.305, *P* < 0.001; Fig. 2C). Skeletal muscle NAMPT correlated positively with VO_2_peak (*r* ^2^ = 0.403, *P* < 0.001; Fig. 2D), lean body mass (*r* ^2^ = 0.139, *P* < 0.01; Fig. 2E) and glucose infusion rate during the last 20 min of the hyperinsulinemic euglycemic clamp (*M*‐value) (*r* ^2^ = 0.172, *P* < 0.01; Fig. 2H). Weak negative correlations were also observed between skeletal muscle NAMPT and total blood cholesterol (*r* ^2^ = 0.113, *P* < 0.05; Fig. 2F) and LDL cholesterol (*r* ^2^ = 0.112, *P* < 0.05; Fig. 2G). Skeletal muscle NAMPT did not correlate with fasting plasma glucose, insulin levels, blood triglycerides, or HDL cholesterol.

### VO_2_peak is a predictor of skeletal muscle NAMPT abundance

A multiple stepwise regression analysis was conducted to determine which among the physiological parameters is/are the predictor(s) of NAMPT protein abundance in skeletal muscle. LDL cholesterol and trunk fat were excluded from the analysis due to collinearity with total cholesterol and body fat, respectively. NAMPT protein abundance in muscle was predicted only by VO_2_peak (*r* ^2^ = 0.391, *F*(1,50) = 35.757, *P* < 0.001; Table 2). Adding *M*‐value, normalized triglycerides, cholesterol, normalized lean body mass, glucose, normalized insulin, normalized HOMA‐IR, BMI, normalized waist‐to‐hip ratio, body fat, HDL cholesterol, body mass, and normalized age in succession did not significantly change the explained variance in NAMPT abundance. The chosen model gave significant coefficients for VO_2_peak as predictor NAMPT protein abundance in muscle [NAMPT abundance = (VO_2_peak)(0.022) + 0.358; Table 3].

**Table 2:** Multiple stepwise regression model of muscle NAMPT abundance and predictor variables

Predictor	*R*	*R* Square	Adjusted *R* Square	Std. Error of the Estimate	Change statistics				
					*R* square change	*F* change	df1	df2	Sig. *F* change
VO_2_peak	0.635	0.403	0.392	0.30352	0.403	35.782	1	53	0.000

Note: The analysis also included *M‐*value, transformed lean body mass, cholesterol, body fat, BMI, and transformed age, which were all excluded in the final model.

**Table 3:** Coefficients of the multiple stepwise regression model

Model	Unstandardized coefficients		Standardized coefficients	*t*	Sig.
	B	Std. Error	Beta		
(Constant)	0.358	0.117		3.053	0.004
VO_2_peak	0.022	0.004	0.635	5.982	0.000

Note: Dependent variable: NAMPT abundance; predictors: (Constant), VO_2_peak.

### Aerobic and resistance exercise training increase human skeletal muscle NAMPT protein

**Figure 3:**
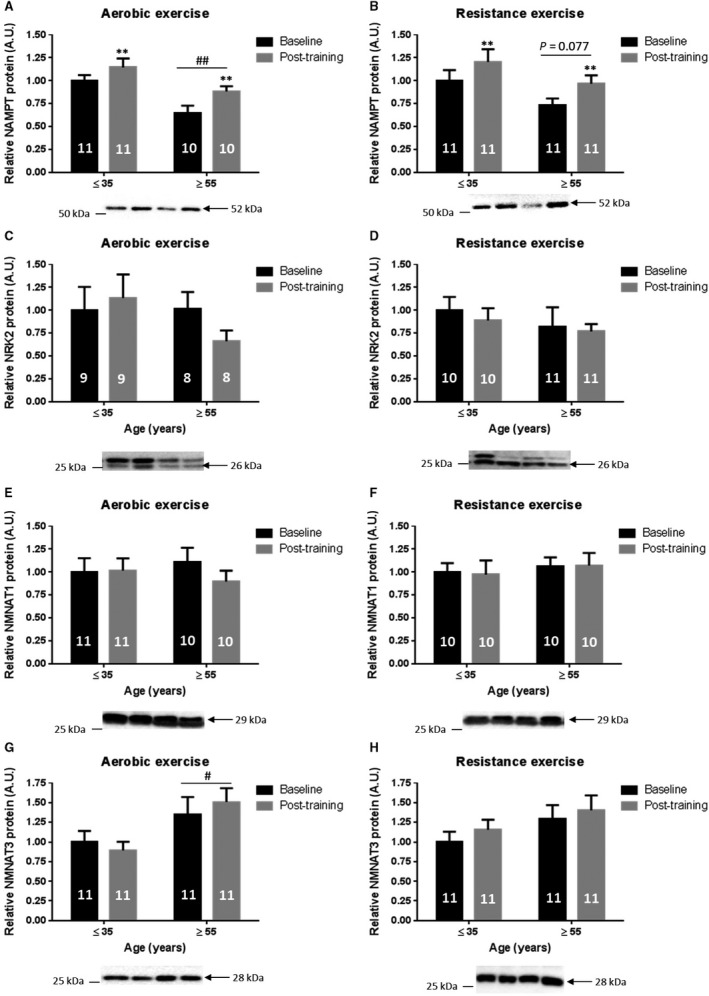
NAMPT protein levels increase with aerobic and resistance exercise training in human skeletal muscle. (A) Skeletal muscle NAMPT in response to aerobic exercise for 12 weeks, ** indicates main effect of training versus baseline (*P* < 0.01) ## indicates main effect of age versus young (*P* < 0.01), no interaction effect. (B) Skeletal muscle NAMPT after 12 weeks of resistance exercise training, ** indicates main effect of training versus baseline (*P* < 0.01), tendency of effect of age *P* = 0.077, no interaction effect. (C) Skeletal muscle NRK2 after aerobic exercise (*n* = 9). (D) Skeletal muscle NRK2 of resistance exercised individuals. (E) Skeletal muscle NMNAT1 in aerobic exercise. (F) Skeletal muscle NMNAT1 in resistance exercise. (G) NMNAT3 in aerobic exercise, # indicates main effect of age versus young (*P* < 0.05). (H) NMNAT3 protein levels in resistance exercise. Number in bar graph indicates number of participants. Statistical test performed: 2‐way RM ANOVA with Tukey as post hoc.

To determine if aerobic or resistance exercise training affected the abundance of the NAD^+^ salvage pathway enzymes, Western blot analyses for NAMPT, NRK2, NMNAT1, and NMNAT3 were performed in human *vastus lateralis* muscle biopsies before and after exercise training in both young (≤35 years old) and older (≥55 years old) participants. In both exercise training groups, skeletal muscle NAMPT protein levels were lower in older compared to young participants, although this was only significant for the group performing aerobic exercise training (Fig. 3A and 3B). Aerobic exercise increased skeletal muscle NAMPT levels ~12% and ~28% in young and older individuals, respectively (effects of training *P* < 0.01, Fig. 3A), whereas resistance exercise training increased muscle NAMPT by ~25% and ~30% in young and older individuals, respectively (effects of training *P* < 0.01, Fig. 3B). With the exception of NMNAT3 in the aerobic exercise cohort, which was higher in the older individuals independent of their training status (Fig. 3G), the other enzymes in the NAD^+^ salvage pathways did not show any significant changes in protein abundance with aging or exercise training (Fig. 3C–F and H).

### eNAMPT level correlates with fasting plasma glucose and insulin, but is unaltered by aerobic or resistance exercise training

**Figure 4:**
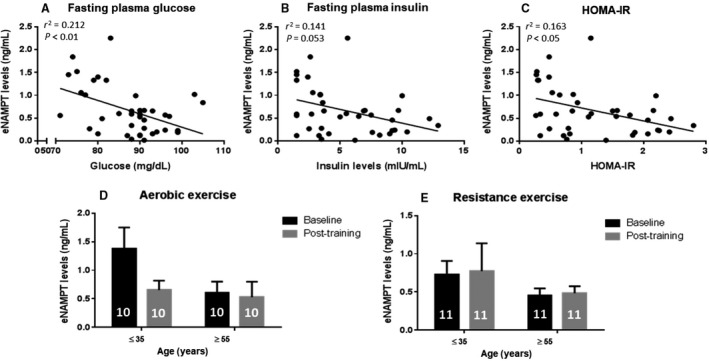
Plasma eNAMPT levels decrease with fasting plasma glucose and insulin levels, but are unaffected by exercise training. (A) Correlation of fasting plasma glucose and eNAMPT levels (*n* = 39, *r* ^2^ = 0.212, *P* < 0.01)*. (B) Correlation of insulin and eNAMPT levels (*n* = 39, *r* ^2^ = 0.141, *P* = 0.053)**. (C) Correlation of HOMA‐IR and eNAMPT levels (*n* = 39, *r* ^2^ = 0.163, *P* < 0.05)**. *Pearson correlation coefficient, *r* was used. Significance of Pearson coefficient was tested using the *t*‐distribution; **Spearman correlation coefficient, *r*_s_ was used; eNAMPT levels in young (≤35 years old) and older (≥55 years old) participants before and after D, aerobic and E, resistance exercise training. Number in bar graph indicates number of participants. Statistical test performed: 2‐way RM ANOVA with Tukey as post hoc.

While skeletal muscle NAMPT levels correlated with age, BMI, body fat percentage, VO_2_peak and lean body mass, none of these parameters correlated significantly with eNAMPT. Nevertheless, eNAMPT levels showed weak, but significant, negative correlations with fasting plasma glucose (*r* ^2^ = 0.2122, *P* < 0.01; Fig. 4A) and HOMA‐IR (*r* ^2^ = 0.163, *P* < 0.05; Fig. 4C) and a marginal correlation with fasting insulin (*r* ^2^ = 0.141, *P* = 0.0525; Fig. 4B). Plasma eNAMPT levels were unaltered by exercise training (Fig. 4D–E).

### Subcutaneous adipose tissue NAD^+^ salvage enzymes are unaffected by HIIT

**Figure 5:**
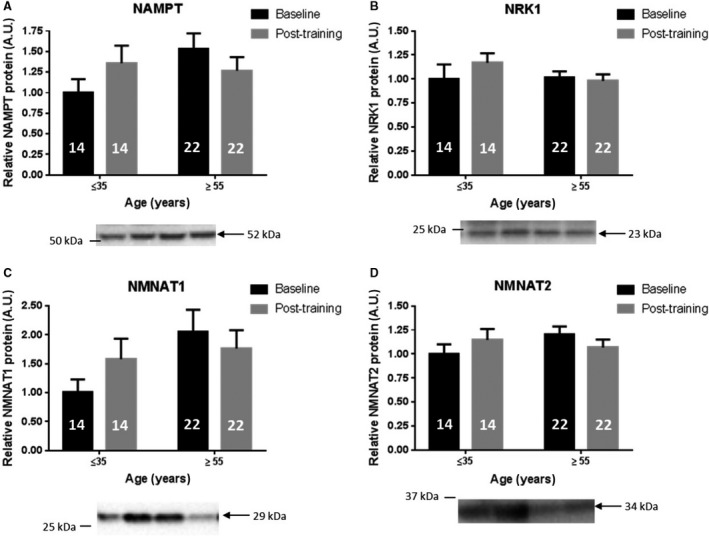
NAD^+^ salvage enzyme levels in adipose tissue of both young and older individuals are unaltered by exercise training. Subcutaneous adipose tissue protein levels of NAD^+^ salvage enzymes at baseline and after high‐intensity interval training (HIIT) for 6 weeks: (A) NAMPT. (B) NRK1. (C) NMNAT1 and (D) NMNAT2. Number in bar graph indicates number of participants. Statistical test performed: 2‐way RM ANOVA with Tukey as post hoc.

To determine if the exercise training‐induced effects on NAMPT in the muscle also take place in adipose tissue, abdominal subcutaneous adipose tissue biopsies were analyzed from young and older subjects before and after HIIT (Larsen et al. 2015; Søgaard et al. 2017). NAMPT, NRK1, NMNAT1, or NMNAT2 abundance was unaltered by HIIT (Fig. 5). Furthermore, age did not correlate with the abundance of any of the individual enzymes (data not shown).

## Discussion

We have previously reported that NAMPT abundance is increased after 3 weeks of one‐legged knee‐extensor exercise training (Brandauer et al. 2013). Furthermore, young athletes have higher protein levels of intramuscular NAMPT compared to nonobese, obese, and diabetic individuals (Costford et al. 2010), and skeletal muscle NAMPT levels are induced by 3 weeks of cycle ergometer training (Costford et al. 2010). NAMPT protein abundance was elevated after 8 weeks of aerobic exercise training in young, but not in older individuals (Johnson et al. 2015). Collectively, these reports support the positive effects of exercise training on skeletal muscle NAMPT levels in younger individuals, but they suggest that aging may compromise the ability of NAMPT to respond to exercise training. Here, we report the effects of different modes of exercise training on protein levels of NAMPT and other NAD^+^ salvage enzymes in human skeletal muscle and subcutaneous adipose tissues from young and older individuals. Our main finding was that, irrespective of exercise modality, 12 weeks of training increased NAMPT abundance in human skeletal muscle of young and older individuals. Moreover, in the older individuals, exercise training was able to completely restore skeletal muscle NAMPT to levels observed in young individuals. Given the role of NAMPT for maintaining the functional capacity (e.g., contractility and oxidative respiration) of skeletal muscle (Frederick et al. 2016; Agerholm et al. 2018), our findings highlight the importance of exercise training as an effective intervention to prevent aging‐associated declines in skeletal muscle function.

Lifelong (>10 years) football training, which consists of a combination of aerobic and resistance training, increases *NAMPT*,* TFAM* and *PGC1α* mRNA levels in skeletal muscle (Mancini et al. 2017) highlighting the possible involvement of the NAD^+^ salvage pathway for exercise training‐induced improvements in mitochondrial function (Alfieri et al. 2015). This link is further supported by findings of increased SIRT3 levels in response to exercise training (Johnson et al. 2015). In animal studies, SIRT3 regulates skeletal muscle metabolism, insulin signaling, handling of reactive oxygen species and the adaptive response to exercise (Palacios et al. 2009; Jing et al. 2011; Vassilopoulos et al. 2014; Cheng et al. 2016). Furthermore, ablation of *Sirt3* in mice compromises mitochondrial complex I and ATP production (Ahn et al. 2008). As SIRT3 activity requires NAD^+^ (Onyango et al. 2002), the elevation of NAMPT in skeletal muscle via exercise training suggests an important role for maintaining muscle function.

We provide evidence that skeletal muscle NAMPT levels correlate negatively with increasing age, BMI, and body fat. These findings corroborate previous reports comparing NAMPT levels in skeletal muscle between young and older individuals (Costford et al. 2010; Johnson et al. 2015). We also confirm earlier reports of a positive correlation between skeletal muscle NAMPT levels and VO_2_peak and glucose infusion rate during a hyperinsulinemic euglycemic clamp (Costford et al. 2010). Multiple stepwise regression analysis showed that VO_2_peak was a significant predictor of skeletal muscle NAMPT levels. The maximal respiratory capacity is affected by body composition and correlates positively with skeletal muscle mass and aerobic performance (Bergh et al. 1978; Anwar and Noohu 2016; Boone et al. 2016; Windisch et al. 2017). This suggests possible links between skeletal muscle NAMPT, NAD^+^ metabolism, and physical activity. Peroxisome proliferator‐activated receptor gamma, coactivator 1 alpha (PGC‐1*α*), a transcriptional cofactor that regulates mitochondrial biogenesis, is deacetylated by SIRT1 and, together with AMPK, is induced by exercise training (Canto and Auwerx 2009; Fernandez‐Marcos and Auwerx 2011). Thus, increased NAD^+^ levels arising from increased NAMPT abundance may lead to activation of sirtuins and PGC‐1*α* (Costford et al. 2010).

While significant effects of exercise training on NAMPT protein abundance were observed for skeletal muscle, we did not find alterations of any of the NAD^+^ salvage enzymes in abdominal, subcutaneous tissues after 6 weeks of cycle ergometer HIIT. A 3‐h bout of exercise has been shown to induce *NAMPT* mRNA expression in adipose tissue up to 6 h after cycling (Frydelund‐Larsen et al. 2007). The whole‐body benefits induced by exercise training include reduced fat mass and enhanced insulin sensitivity. However, the positive effects of exercise training on adipose tissue may be depot‐specific. Visceral and subcutaneous adipose tissues show varying degrees of insulin resistance during obesity and type 2 diabetes (Bonora 2000; Frayn 2000; Hsieh et al. 2014). A study of 16 obese subjects who underwent Roux‐en‐Y gastric bypass surgery, provided evidence of similar NAMPT protein levels in subcutaneous adipose tissue of insulin‐sensitive and insulin‐resistant subjects (Xu et al. 2012). However, significant differences were observed in NAMPT levels of visceral fat depots (Xu et al. 2012). In a separate study with obese and non‐obese individuals, *NAMPT* mRNA levels were unaltered in subcutaneous adipose tissue depots (Chang et al. 2010). Furthermore, subcutaneous and visceral adipose *NAMPT* mRNA levels were not correlated with clinical features including BMI, waist circumference, fasting glucose and insulin, and HOMA‐IR (Chang et al. 2010). The potential adipose depot‐specific effects on NAMPT protein abundance in response to exercise training is an interesting topic for future studies.

Apart from NAMPT, none of the other enzymes in the NAD^+^ salvage pathway (i.e., NRK and NMNAT) were altered by exercise training in skeletal muscle. These results further highlight the importance of NAMPT in regulating NAD^+^ pools of skeletal muscle. However, the mechanism by which exercise training promotes NAMPT abundance in skeletal muscle is unclear. Our previous work suggested that AMPK controls NAMPT levels in mouse skeletal muscle through a posttranscriptional mechanism (Brandauer et al. 2013). However, the *Nampt* promoter is regulated by the circadian core clock machinery (Ramsey et al. 2009) and SIRT1 (Nakahata et al. 2009), and exercise alters mRNA expression of clock genes in the skeletal muscle in both mice (Wolff and Esser 2012; Yasumoto et al. 2015) and humans (Zambon et al. 2003). Therefore, whether the increased NAMPT protein content in human skeletal muscle after aerobic and resistance training is a consequence of transcriptional activity of the clock machinery or another posttranscriptional mechanism warrants further investigation. Interestingly, NAMPT in human pulmonary artery endothelial cells appears involved in the pathophysiology of acute respiratory distress syndrome, and mechanical stress‐inducible regions have been identified in the *NAMPT* promoter upstream of the transcription start site (Sun et al. 2014). These mechanical stress‐inducible regions are involved in epigenetic changes in the regulation of *NAMPT* expression (Adyshev et al. 2014; Elangovan et al. 2016). Such a mechanism could potentially account for the exercise training‐induced increase in NAMPT in contracting skeletal muscle in response to the physical activity.

eNAMPT correlates with increased adiposity in obesity and type 2 diabetes (Retnakaran et al. 2008; Kocelak et al. 2015; Owczarek et al. 2016). However, eNAMPT levels did not correlate with age, consistent with a study with 163 individuals covering an age‐range from 24 to 86 years of age (Berndt et al. 2005). While the previous study reported a positive correlation between eNAMPT, BMI, and percent body fat, we did not observe this relationship in our cohort. The difference between these studies might be due to the inclusion of participants with type 2 diabetes, glucose intolerance, and obesity, which are not represented in our study. In contrast, we found weak negative correlations between eNAMPT and fasting plasma glucose, insulin, and HOMA index. Moreover, 12 weeks of exercise training did not affect eNAMPT levels in serum. Similar results were observed in a comparable study involving 40 older women (≥55 years old) with type 2 diabetes separated into distinct groups including untrained, aerobic trained, resistance trained, and a combined training group for 12 weeks (Mehdizadeh et al. 2016). However, other studies have provided evidence for increased (Jorge et al. 2011) or decreased (Haus et al. 2008; Seo et al. 2011) eNAMPT levels after 12 weeks of exercise training. Collectively, these findings suggest that physical activity is unlikely to be a primary determinant of circulating eNAMPT levels.

In conclusion, we report that aerobic and resistance exercise training increases NAMPT protein abundance in skeletal muscle of humans independent of age. Conversely, skeletal muscle NRK2 and NMNAT1/3 levels, as well as eNAMPT abundance and adipose tissue levels of NAD^+^ salvage enzymes were unaffected by exercise training. These findings indicate that increasing NAMPT may be a critical and important aspect of the improvement in muscle function seen with exercise training.

## Conflict of Interest

The authors report no conflict of interest.
